# Tensile Behavior and Failure Mechanism of Bamboo Fiber Bundle and Its Scrimber Under Different Strain Rates

**DOI:** 10.3390/ma18112550

**Published:** 2025-05-29

**Authors:** Kai Zhang, Haoran Xia, Lizhi Xu, Shengbo Zhou, Li Gao, Gong Zuo, Xiaotao Zhang, Quan Li

**Affiliations:** 1 School of Civil Engineering and Architecture, Suqian University, Suqian 223800, China; 2 Department of Architecture, Graduate School of Engineering, The University of Tokyo, Tokyo 113-8654, Japan; 3 School of Mechanical Engineering, Nanjing University of Science and Technology, Nanjing 210094, China

**Keywords:** bamboo fiber bundle, bamboo scrimber, strain rates, tensile behaviors, failure modes

## Abstract

In this study, bamboo fiber bundles were directly extracted from raw bamboo material to fabricate reconstituted bamboo using the traditional hot-pressing method. The tensile behaviors and failure mechanisms of both the bamboo fiber bundle and its bamboo scrimber under various strain rates (quasi-static, 350/s, 950/s and 1700/s) were investigated by the SHTB system (split-Hopkinson tensile bar, high-speed camera and digital image correlation method). The results showed that the bamboo scrimber exhibited an obvious positive strain rate effect. The ultimate tensile strength of the bamboo scrimber at a strain rate of 1700/s was close to 200 MPa, but it was only about 80 MPa under quasi-static loading. This experimental result was further validated by the tensile behaviors of single bamboo fiber bundles at different strain rates (quasi-static, 300/s, 700/s and 1500/s). In addition, as the strain rate increased, the fracture surface of the bamboo changed from a linear shape to a discontinuous folded shape.

## 1. Introduction

With the increasing interest in sustainable green materials and renewable resources, more and more researchers are paying attention to the processing and utilization of natural bamboo materials [1,2,3]. Bamboo, as a natural resource, is widely planted in many countries and regions and has advantages such as rapid growth, high strength, lightweight, carbon sequestration and land rehabilitation, thus attracting special attention from researchers and engineers [4,5]. However, due to the inherent size and shape characteristics of bamboo itself, raw bamboo material cannot be directly used in structural engineering [1]. Therefore, a recombined bamboo (bamboo scrimber) technique was developed in recent years. Bamboo scrimber is a concept of using crushed bamboo fiber bundles for reprocessing and reassembly with resins, aimed at creating new bamboo products and materials. By using methods such as slicing, softening, pressing, drying and bonding bamboo, the structure and shape of bamboo can be changed, thereby improving its performance and expanding its application range [6]. The new bamboo material possesses exceptional qualities, including an impressive bamboo utilization ratio exceeding 80%, excellent mechanical properties and stable performance, making it a potential alternative to traditional wood and plastic in structural engineering applications.

The latest research on bamboo scrimber include the optimization of the preparation process, surface coating, physical–chemical treatment, machining, mechanical behaviors, thermal property, etc. [7,8,9,10,11,12,13]. Liang et al. [7] developed a one-time drying method to optimize the efficiency of bamboo scrimber preparation and drastically reduced the energy consumption during the manufacture process. Peng et al. [8] obtained a kind of unique bamboo scrimber by immersing bamboo bundles in a P-N-B solution, and the bamboo scrimber showed excellent flame-resistance characteristics. Yuan et al. [9] used a methyl silicone oil treatment to modify bamboo scrimber and analyzed the surface morphology, swelling properties, chemical structure and contact angle to study the treatment effectiveness. For the application of bamboo scrimber in the field of structural load-bearing components, its mechanical properties are the most critical indicators. Therefore, in the past 10 years, a large amount of research has focused on the mechanical behaviors and failure mechanisms of bamboo scrimber [10,11,12,13]. Li et al. [10] developed a new preparation method and proposed three different stress–strain relationship models to study the basic mechanical behaviors of bamboo scrimber. Lei et al. [11] studied the specific mechanical performance of bamboo scrimber under extreme weather conditions. The results showed significant structural changes of bamboo scrimber after different hydrothermal drying–hydrothermal cycle aging. Wang et al. [12] studied the compressive properties and failure modes of bamboo scrimber in three directions under various strain rates by the split-Hopkinson pressure system. The results showed that the strength and stiffness of bamboo scrimber could be affected by different sizes and shapes. Guan et al. [13] investigated the effects of pre-tension force and strain rate on the mechanical properties of bamboo scrimber. The experimental results showed that the mechanical behaviors and fracture mechanisms of bamboo scrimber were significantly influenced by both the pre-tension force and loading rate.

Despite so many studies on the mechanical properties of bamboo scrimber, bamboo materials are currently more commonly used in non-load-bearing components such as outdoor flooring, garden fences, furniture decoration and the like. As the Kingdom of Bamboo, the utilization rate of bamboo in China is only around 20% [14]. However, in countries like the United States, Germany, and others, bamboo materials are utilized in building materials for constructing small cottages. In particular, bamboo scrimber has shown the potential for broader applications in the future, such as highway guardrails, transportation vehicles, sports equipment and so on [15]. The application of reconstituted bamboo material in these fields requires excellent impact resistance properties. Therefore, it is necessary to conduct experimental and theoretical research on the dynamic mechanical behaviors of reconstituted bamboo. However, to the best of our knowledge, most of the mechanical research on reconstituted bamboo has focused on its quasi-static mechanical properties. Very limited studies have investigated the dynamic mechanical behaviors of bamboo fiber bundles and the corresponding bamboo scrimber materials. There is still extensive research needed on how bamboo fiber bundles and their composites respond under high strain rates.

Therefore, in this paper, bamboo scrimber was fabricated by 90 wt.% mesh-like bamboo fiber bundles and 10 wt.% phenolic resin using the commonly used hot-pressing method. The dynamic tensile properties of bamboo fiber bundles and their bamboo scrimber were comparatively studied by the SHTB system. Meanwhile, the failure mechanisms of the bamboo fiber bundles and bamboo scrimber under different strain rates were analyzed by capturing real-time images with a high-speed camera. This study may offer valuable perspectives that could broaden the application of bamboo scrimber materials in the dynamic impact engineering field.

## 2. Experimental Section

### 2.1. Materials Preparation

**Figure 1:**
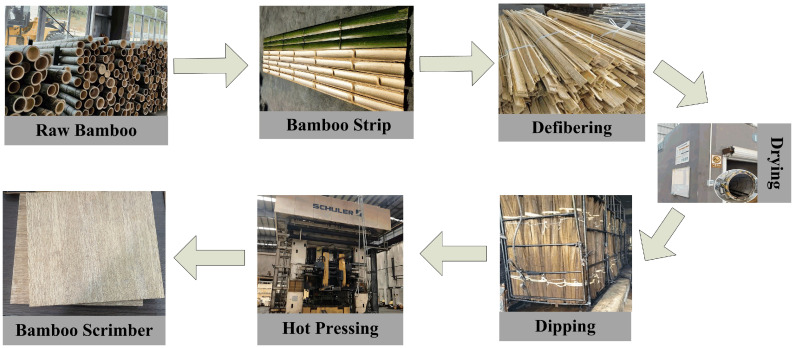
Recombinant bamboo preparation process diagram.

The preparation method of the bamboo fiber bundles tested in this article was as follows: the bamboo was longitudinally divided, and the outer green and inner yellow parts of the raw bamboo were removed; then, it was thinned into a mesh structure, and finally the required bamboo fiber bundles were cut with knife. It should be noted that in order to avoid significant experimental errors, the bamboo fiber bundles cut should exclude the bamboo nodes. As shown in Figure 1, the unit material of bamboo scrimber is called bamboo strands or bamboo fiber bundles, which are usually mesh-like and are made by cutting, slicing, removing the green and yellow parts of raw bamboo and rolling and separating bamboo materials. Subsequently, the bamboo fiber bundles undergo drying, gluing, further drying, shaping and hot pressing to form sheet-like or square bamboo materials, namely reconstituted bamboo or bamboo scrimber. The key processes in the production of reconstituted bamboo include bamboo strand production, gluing, drying, loading and shaping, which require specialized equipment for processing and production. The type of bamboo used in our study was mature moso bamboo, extracted from Fujian Province, and we were kindly supported by Bamboo Technology Co., Ltd. (Yongan, China). The adhesive was a commonly used phenolic resin. The bamboo scrimber material consisted of 90 wt.% bamboo fiber bundles and 10 wt.% phenolic resin in this study.

### 2.2. Quasi-Static Tensile Test

**Figure 2:**
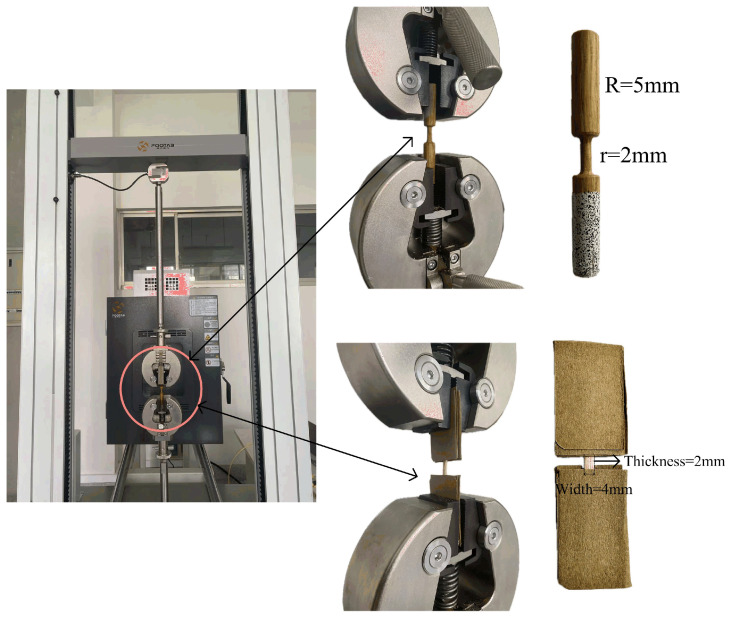
Quasi-static tensile test based on universal Instron testing machine.

Figure 2 shows the quasi-static tensile tests of a single bamboo fiber bundle and its bamboo scrimber. To ensure the accuracy of the experimental data, it is crucial to prevent sample failure caused by stress concentration at the clamping locations. Therefore, at the clamping locations of bamboo fiber bundles, we used thick cardboard to ensure that the testing sample could break near the center when subjected to tensile loading. The tensile test was conducted at room temperature, with the fixture operating at a velocity of 2 mm/min. To mitigate size effects, the dimensions of the single bamboo fiber bundle and bamboo scrimber under the quasi-static test were same as the dynamic ones. The dimensions of the testing samples are presented in Figure 2. Meanwhile, a camera was used to record the progressive failure of testing specimens under quasi-static loading. Finally, for the reliability of the experiment, at least three parallel tests should be conducted for each sample under various strain rates.

### 2.3. Dynamic Tensile Test

**Figure 3:**
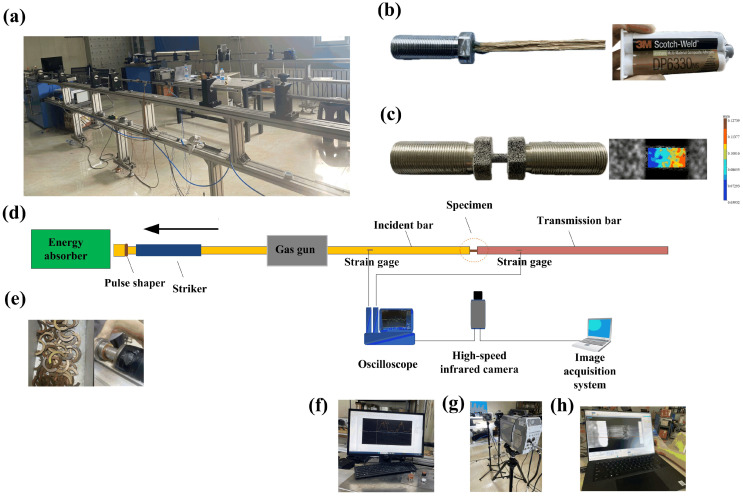
Split-Hopkinson tensile bar system (SHTB) with a high-speed camera: (**a**) SHTB equipment, (**b**) fixture of single bamboo fiber bundle, (**c**) fixture of bamboo scrimber with DIC method, (**d**) schematic diagram of SHTB system, (**e**) copper pulse shapers, (**f**) oscilloscope with dynamic strain indicator, (**g**) high-speed camera and (**h**) image acquisition system.

As shown in Figure 3, the split-Hopkinson tensile bar (SHTB) testing system is commonly employed in experimental research focused on polymer composites subjected to different strain rates [16,17,18]. Figure 3a–h display the testing equipment, the homemade fixture, a testing specimen with speckle patterns, copper-made pulse shapers, the oscilloscope, dynamic strain gauges, the high-speed camera and the image acquisition system, respectively.

To securely fix the test specimen in the fixture and prevent it from slipping during testing, Urethane Multi-Material Composite Adhesive (3M Scotch-Weld DP6330_NS,_ from 3M company, St. Paul, MN, USA) was used in the experiment. After dynamic tensile tests, it is necessary to carefully observe whether the sample has any delamination phenomenon to ensure the reliability of the experimental data.

The striker, incident bar and transmitted bar have lengths of 400 mm, 3000 mm and 2000 mm, correspondingly. Each bar has a diameter of 16 mm and is composed of an aluminum alloy, characterized by a Young’s modulus of E = 70 GPa and a density of ρ = 2.8 g/cm^3^. During the impact test, the striker impacts the end of the incident bar, generating a tensile wave. The high-speed camera activates upon detecting the input signal from the strain gauge and oscilloscope. It is important to note that due to the low tensile strength and fracture strain of the bamboo fiber bundles and their bamboo scrimber, the digital image correlation method was also used to confirm the strain of the testing specimen. Stress and strain were computed using the one-dimensional elastic wave theory [19]. The engineering strain ($\varepsilon_{E}\left(t\right)$) and engineering stress ($\sigma_{E}\left(t\right)$) of the specimen were determined based on the following equations [20]:

(1) $$\varepsilon_{E}\left(t\right)=-\frac{2C_{0}}{L_{s}}\int_{0}^{t}\varepsilon_{R}(t)dt$$

(2) $$\sigma_{E}\left(t\right)=E_{0}\frac{A_{0}}{A_{s}}\varepsilon_{T}(t)$$

In the equations presented, $E_{0}$, $A_{0}$ and $C_{0}$ denote the Young’s modulus, the cross-sectional area of the incident bar and the stress wave velocity, respectively. $L_{s}$ and $A_{s}$ represent the length and cross-sectional area of the specimen, respectively; $\varepsilon_{R}(t)$ signifies the tensile strain history recorded in the incident bar, while $\varepsilon_{T}(t)$ indicates the tensile strain history of the transmitted bar as measured by a high-precision semiconductor strain gauge. The average strain rate of the test specimen was determined using a high-speed camera and digital image correlation (DIC) techniques. To guarantee the experiment’s reliability, it was necessary to obtain at least two sets of repeated experimental results for the same specimen.

## 3. Results and Discussion

### 3.1. Tensile Behaviors of Single Bamboo Fiber Bundle and Its Scrimber Under Quasi-Static Loading Condition

**Figure 4:**
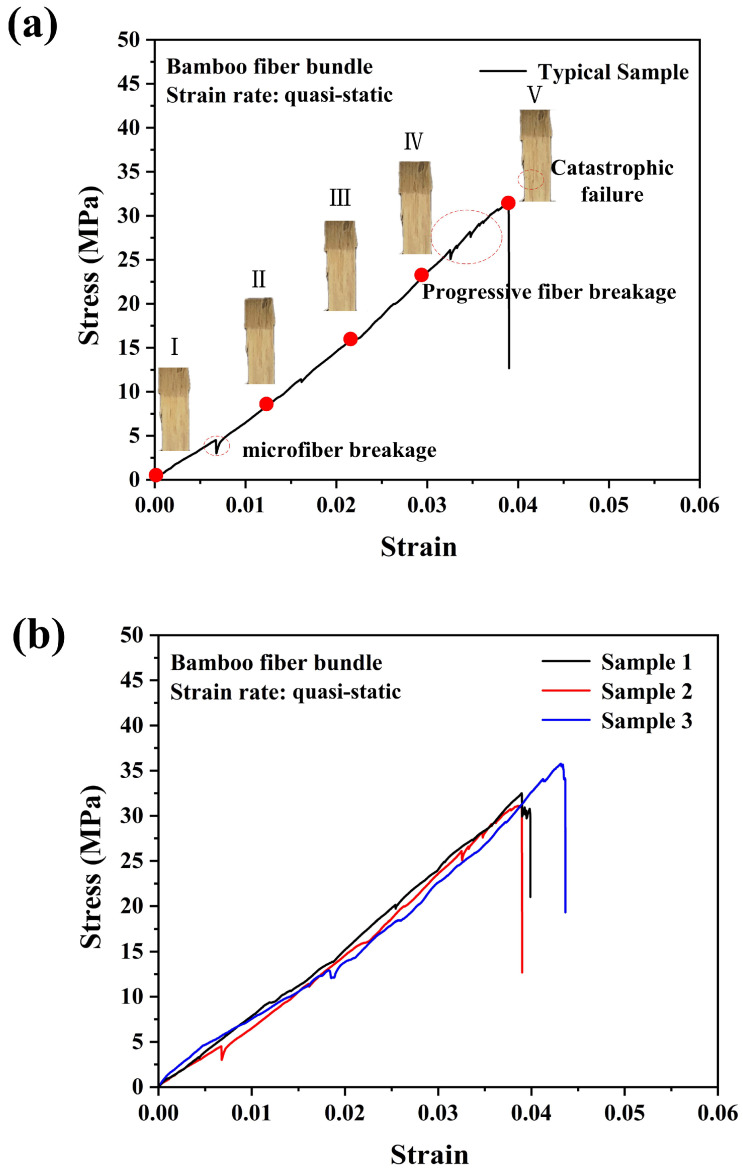
Quasi-static tensile stress–strain curves and progressive failure of single bamboo fiber bundle (**a**) and repeatability of the experiment (**b**).

Figure 4a,b present the tensile behaviors and repeatability of results of the single bamboo fiber bundle under quasi-static loading. Firstly, it should be noted that, as shown in Figure 1, the single bamboo fiber bundle referred to in this paper is actually macro-bamboo fiber that has been directly fiberized from bamboo strips and manually segmented. A single bamboo fiber bundle could also be further divided into more small bamboo fiber strands. The single bamboo fiber bundle exhibited typical linear elastic behavior as a whole. After reaching the ultimate tensile stress, the bamboo fiber bundle immediately underwent brittle and catastrophic failure. However, as shown in Figure 4a, during the bamboo fiber stretching process, there were still some tiny bamboo fibers that broke earlier, which could be observed between stage I and stage II of the stress–strain curve. And when a single bamboo fiber approached final failure, a large number of tiny fibers began to break and failed, which could be seen between stage IV and stage V of the stress–strain curve. Table 1 presents detailed data of the ultimate tensile strength of the single bamboo fiber and its bamboo scrimber under different strain rates. For the quasi-static condition, the ultimate tensile strength of the single bamboo fiber was around 32 MPa. It should be clarified that the bamboo fiber bundles used in this experiment excluded the bamboo nodes, which ensured consistency in the experimental results [21].

**Figure 5:**
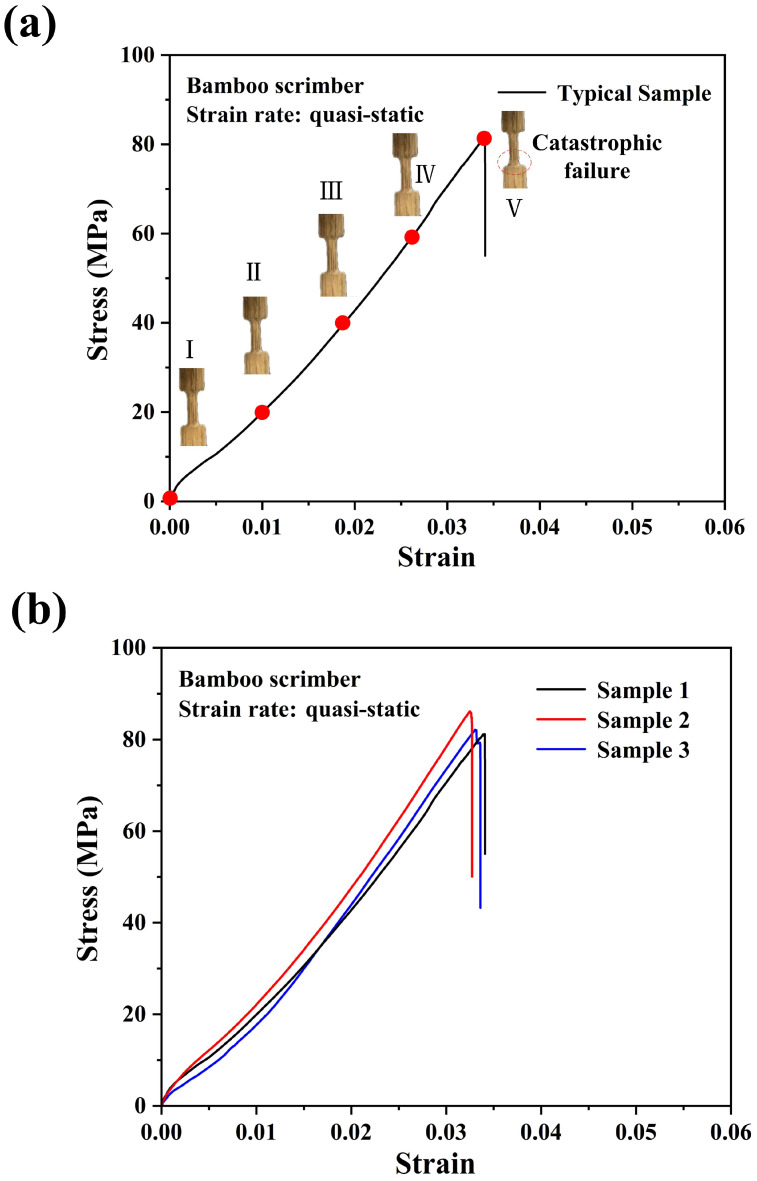
Quasi-static tensile stress–strain curves and progressive failure of bamboo scrimber (**a**) and repeatability of the experiment (**b**).

Figure 5a,b presents the tensile behaviors and repeatability of the results of the bamboo scrimber under quasi-static loading. In comparison, the bamboo scrimber displayed a smooth linear elastic tensile behavior. The ultimate tensile strength of the bamboo scrimber was around 82 MPa. Li et al. [6] studied the size effect on the tensile strength of bamboo scrimber with the consideration of density. The results showed that the ultimate tensile strength of the bamboo scrimber was around 110 MPa, which was approximately 34% higher than the experimental results in this study. Wang et al. [22] studied the tensile mechanical properties and failure mechanism of bamboo scrimber under different strain rates. The results showed that the ultimate tensile strength of bamboo scrimber under quasi-static loading was also around 110 MPa. The tensile strength of the bamboo scrimber was relatively low, which may be due to the size of the sample used in our study. In addition, the mechanical behavior of bamboo itself may vary due to its different types, growth years, growth locations and other factors [23]. Compared with the bamboo fiber bundle, the bamboo scrimber exhibited significantly higher tensile strength due to the preparation process of recombinant bamboo, which densified the bamboo fiber bundles, thereby increasing the density and strength of the composite material.

### 3.2. Tensile Behaviors of the Single Bamboo Fiber Bundle and Its Bamboo Scrimber Under SHTB Loading

#### 3.2.1. Stress Equilibrium in SHTB Test

**Figure 6:**
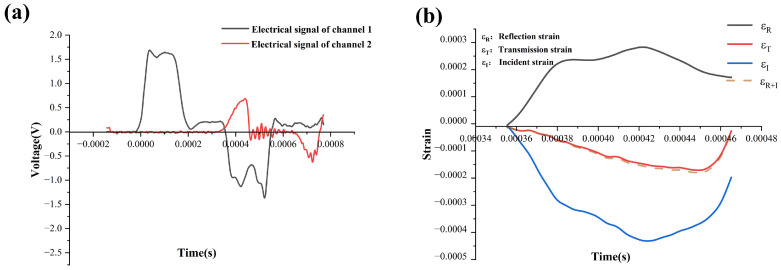
Example of the split-Hopkinson tensile bar (SHTB) original signal for a testing sample: (**a**) original signal recorded by an oscilloscope and (**b**) stress equilibrium in specimen.

It is widely acknowledged that achieving stress equilibrium at both ends of the specimen plays a crucial role in ensuring the reliability of experimental outcomes in an SHTB (split-Hopkinson tensile bar) test [17]. A typical experimental signal depicting the incident wave and reflected wave is illustrated in Figure 6. The Hopkinson tensile test is based on the one-dimensional elastic wave theory, and in the Hopkinson tensile test (SHTB), waves are indeed reflected through electrical signals. In the experiment, the propagation of stress waves in the rod was captured by strain gauges attached to the rod. Figure 6a shows the electrical signals measured by channel 1 and channel 2 of the dynamic strain gauge, which were used to convert the corresponding strain values. As shown in Figure 6b, after superimposing the incident strain ε_I_ and reflected strain ε_R_, it was found that they matched well with the transmission strain ε_T_. This result suggested that the dynamic tensile test was conducted under favorable stress equilibrium conditions. Consequently, the stress–strain curves derived subsequently were deemed accurate and dependable.

#### 3.2.2. Dynamic Tensile Behaviors of the Bamboo Fiber Bundle and Its Scrimber

**Figure 7:**
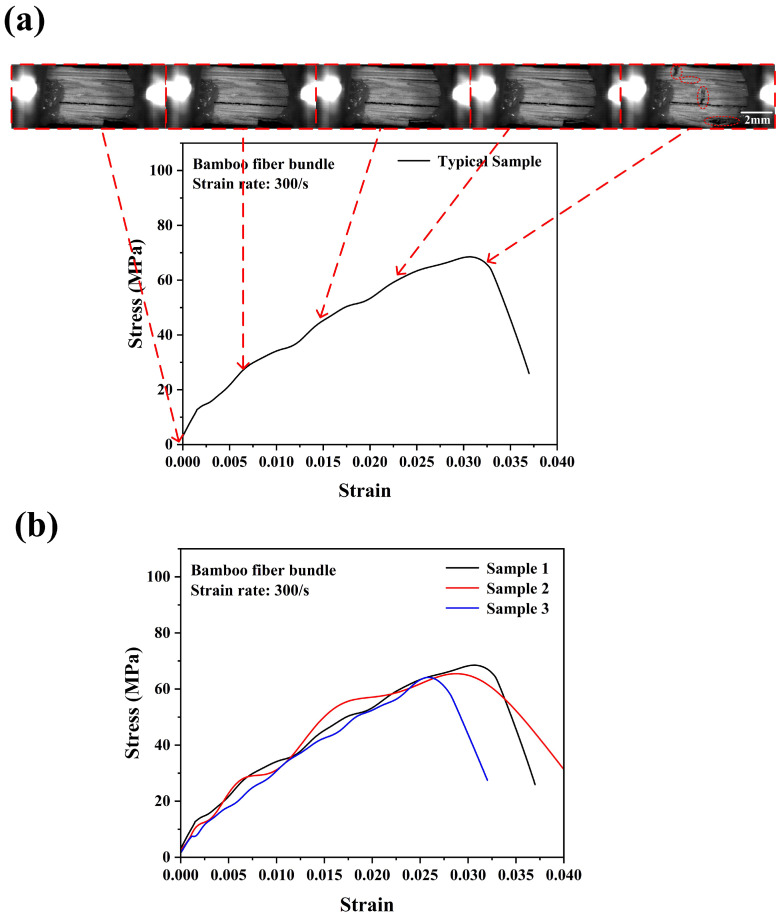
Dynamic tensile stress–strain curve with real-time images of the bamboo fiber bundle (**a**) and consistency of experimental results (**b**) at a strain rate of 300/s.

As shown in Figure 7, under a dynamic tensile load with a strain rate of 300/s, the bamboo fiber bundle exhibited obviously higher ultimate tensile strength compared to the quasi-static condition, with an average value of ~66 MPa. These results indicated that the bamboo had a positive strain rate effect. Figure 7b showed the repeatability of the tensile stress–strain curves of the bamboo fiber bundles under a strain rate of 300/s. Compared to quasi-static loading, the tensile stress–strain curve of the bamboo fiber bundles exhibited significant oscillations with the increase in stress, and when the fibers reached their maximum stress, the stress values did not instantly decrease to zero. In other words, the bamboo fiber bundles did not exhibit perfect linear elastic behavior at a higher strain rate. However, this may be mainly due to the characteristics of the SHTB test itself [24]. Some studies have shown that SHTB experiments have high accuracy in measuring the strength of samples, but the elastic moduli of the materials are not accurate enough [25,26,27]. Therefore, this paper does not provide the elastic moduli of materials at different strain rates. Figure 7a shows the progressive failure image of the bamboo fiber bundle under dynamic tensile loading. It could be observed that the bamboo fiber bundles were composed of many small bamboo fiber filaments. When the bamboo fiber bundle ultimately failed, these bamboo fiber filaments would nearly simultaneously undergo breakage. However, under the quasi-static condition, some tiny bamboo fibers would break earlier before the catastrophic failure of the bamboo fiber bundle. This was mainly due to the slow loading speed, which provided sufficient time for the stress transmission in the testing sample. Due to its natural uneven properties, some parts of the bamboo fiber bundle have weaker mechanical properties, making it prone to premature fracture and failure [23]. And when the strain rate increased, the bamboo fiber bundle would fail in a very short time, thus showing the characteristic of almost simultaneous breakage of bamboo fiber filaments. As expected, the fracture strain of the bamboo fiber bundle under a strain rate of 300/s was smaller in comparison with the quasi-static condition. This indicated that the bamboo fiber bundle exhibited more brittle tensile behavior under a higher strain rate.

**Figure 8:**
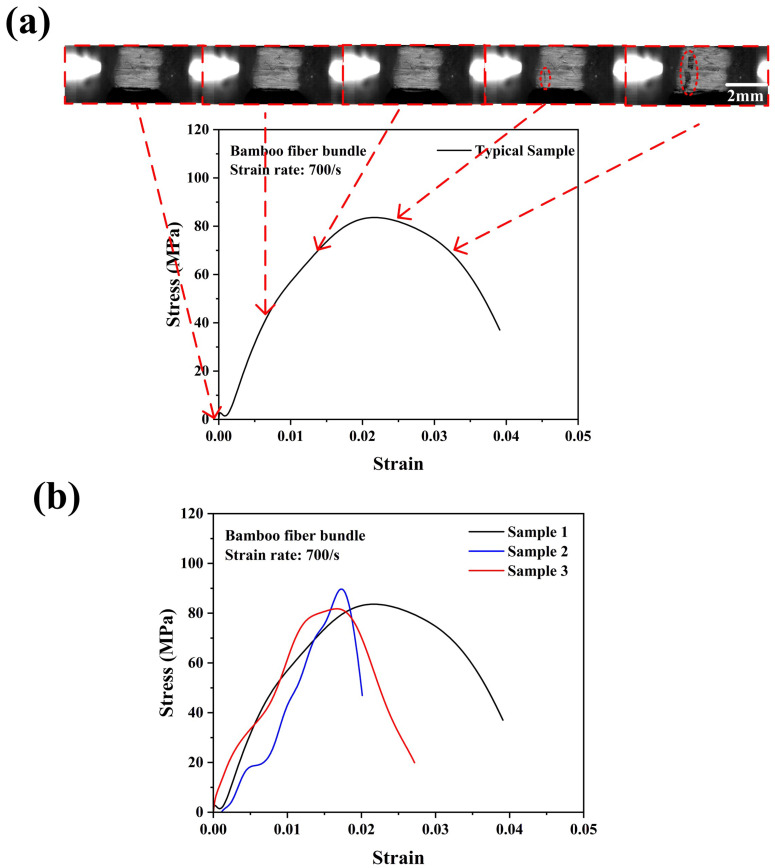
Dynamic tensile stress–strain curve with real-time images of the bamboo fiber bundle (**a**) and the consistency of the experimental results (**b**) at a strain rate of 700/s.

**Figure 9:**
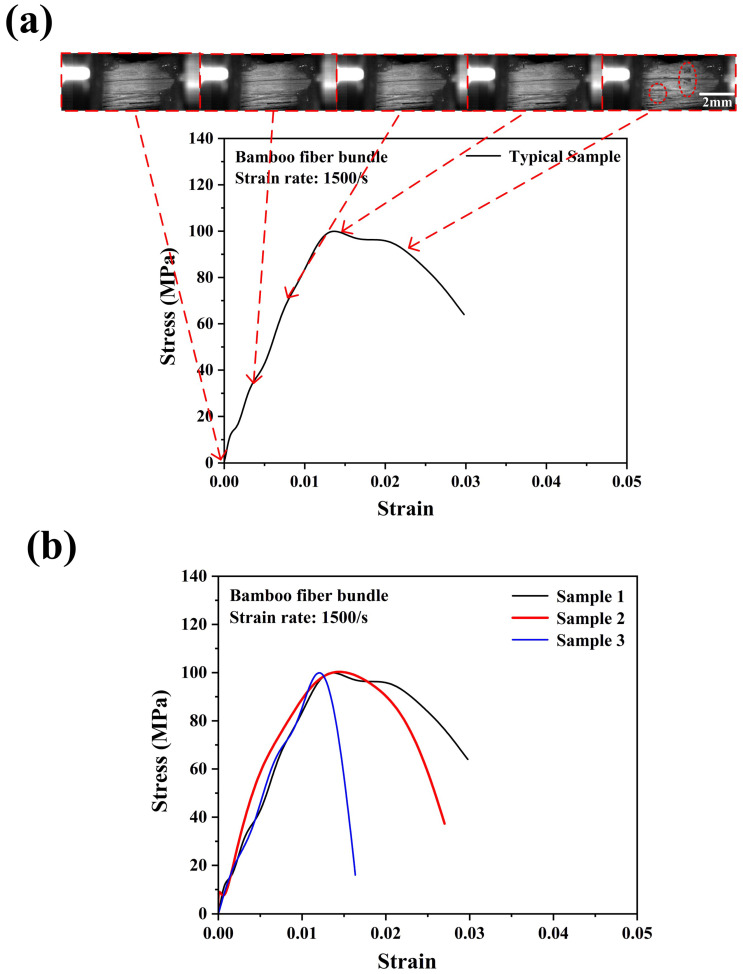
Dynamic tensile stress–strain curve with real-time images of the bamboo fiber bundle (**a**) and the consistency of the experimental results (**b**) at a strain rate of 1500/s.

Figure 8a,b show the typical failure process of a bamboo fiber bundle and the repeatability of the tensile stress–strain curves of bamboo fiber bundles under a strain rate of 700/s. The patterns of the stress–strain curves of bamboo fiber bundles at a strain rate of 700/s varied greatly. However, the ultimate tensile strengths of the bamboo fiber bundles still exhibited good repeatability. This may also be due to the characteristics of the SHTB experiments. In addition, with the increase in the strain rate, the bamboo fiber bundle showed a higher tensile strength of 85 MPa. Compared with quasi-static and 300/s conditions, the bamboo fiber bundle reached the highest tensile stress at a lower strain at a strain rate of 750/s. Meanwhile, it could be observed from Figure 8a that the damage locations of bamboo fibers were not at the two ends of the fixture where the stress concentration could occur, indicating that the experimental results were reliable. Similarly, as the strain rate continued to increase to 1500/s, as shown in Figure 9, the stress–strain curves of the bamboo fiber bundles exhibited a similar approximate linear elastic behavior, indicating that as the strain rate increased, the bamboo fiber bundles did not generate ductile failure but rather become more brittle. The average maximum stress of the bamboo fiber bundle at a strain rate of 1500/s is about 100 MPa. Similarly, the bamboo fiber bundles even failed at strains below 0.15, which further proved that the bamboo fiber bundles exhibited a positive strain rate effect.

**Figure 10:**
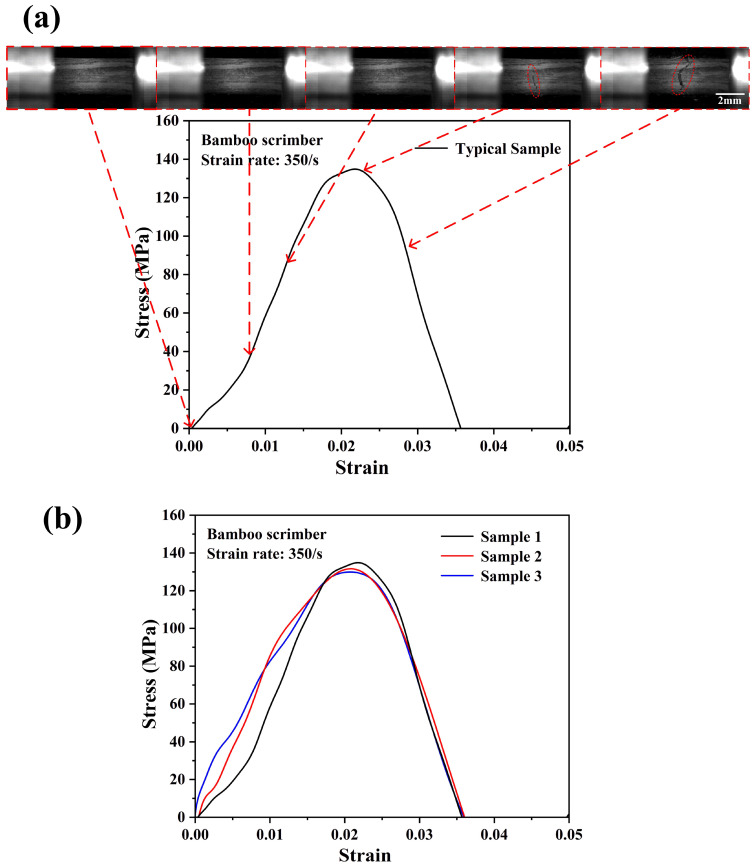
Dynamic tensile stress–strain curve with real-time images of the bamboo scrimber (**a**) and the consistency of the experimental results (**b**) at a strain rate of 350/s.

Figure 10a,b show the typical failure process of bamboo scrimber and the repeatability of the tensile stress–strain curves of the testing specimen under a strain rate of 350/s.

**Figure 11:**
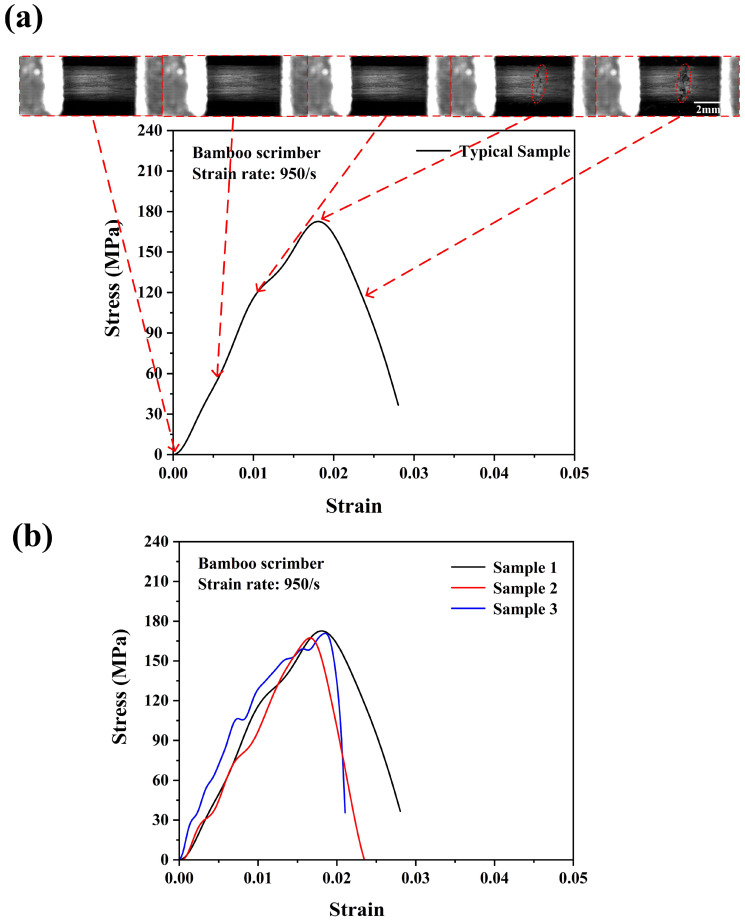
Dynamic tensile stress–strain curve with real-time images of the bamboo scrimber (**a**) and the consistency of the experimental results (**b**) at a strain rate of 950/s.

**Figure 12:**
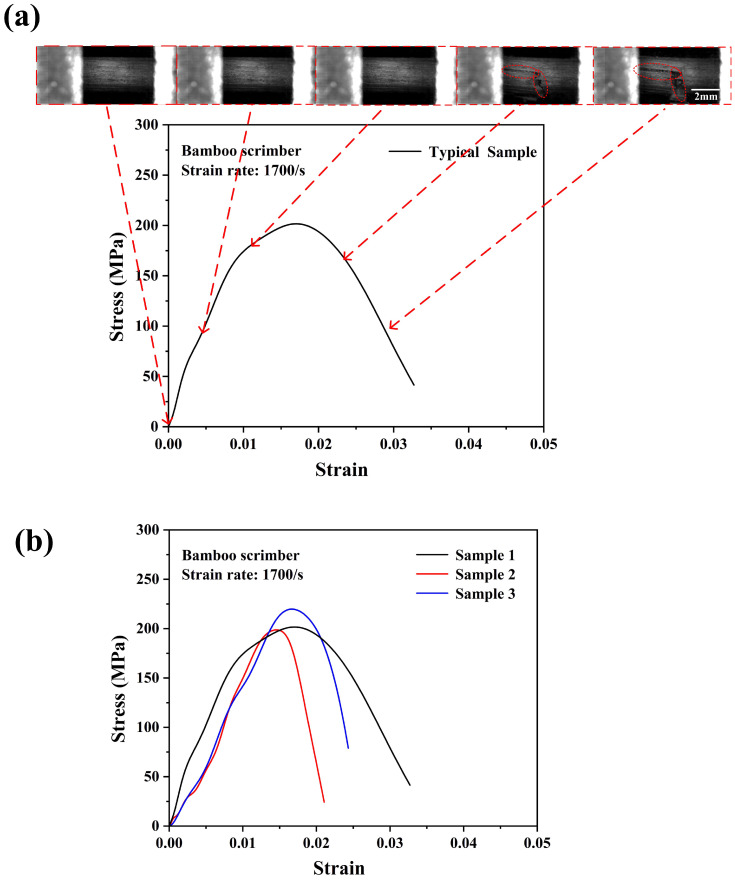
Dynamic tensile stress–strain curve with real-time images of the bamboo scrimber (**a**) and the consistency of the experimental results (**b**) at a strain rate of 1700/s.

The strain rate under the dynamic tensile loading conditions could not be precisely controlled at a determined value due to the SHTB test characteristics. However, when the strain rates were not significantly different, there would be no significant differences in the stress–strain behaviors of the testing specimen [26]. The ultimate stress of the bamboo scrimber under a strain rate of 350/s was ~131 MPa, which was about 60% higher compared with the quasi-static condition. This result indicated that the bamboo scrimber also had an obvious positive strain rate effect. The bamboo scrimber consisted of 90 wt.% highly compacted bamboo fiber bundles and 10 wt.% phenolic resin. On the one hand, through the above experimental results, the bamboo fiber bundle itself exhibited an obvious positive strain rate effect. On the other hand, some studies have shown that phenolic resins also exhibit a positive strain rate effect [28,29]. Similarly, as shown in Figure 11a,b, the average ultimate strength of the bamboo scrimber was ~170 MPa, and the testing specimen broke at a smaller strain compared with that of lower strain rates. Table 1 presents the values of the ultimate tensile strengths of bamboo scrimber under different strain rates. As expected, from Figure 12a,b, the ultimate strength of the bamboo scrimber further increased to ~206 MPa. Lastly, it should be noted that some testing specimens may fail near the fixture port, but the results exhibited no obvious differences in the stress–strain curves. This indicated that the data obtained from the SHTB test were reliable.

### 3.3. Rate Dependence of Tensile Properties of Bamboo Fiber Bundle and Its Bamboo Scrimber

**Figure 13:**
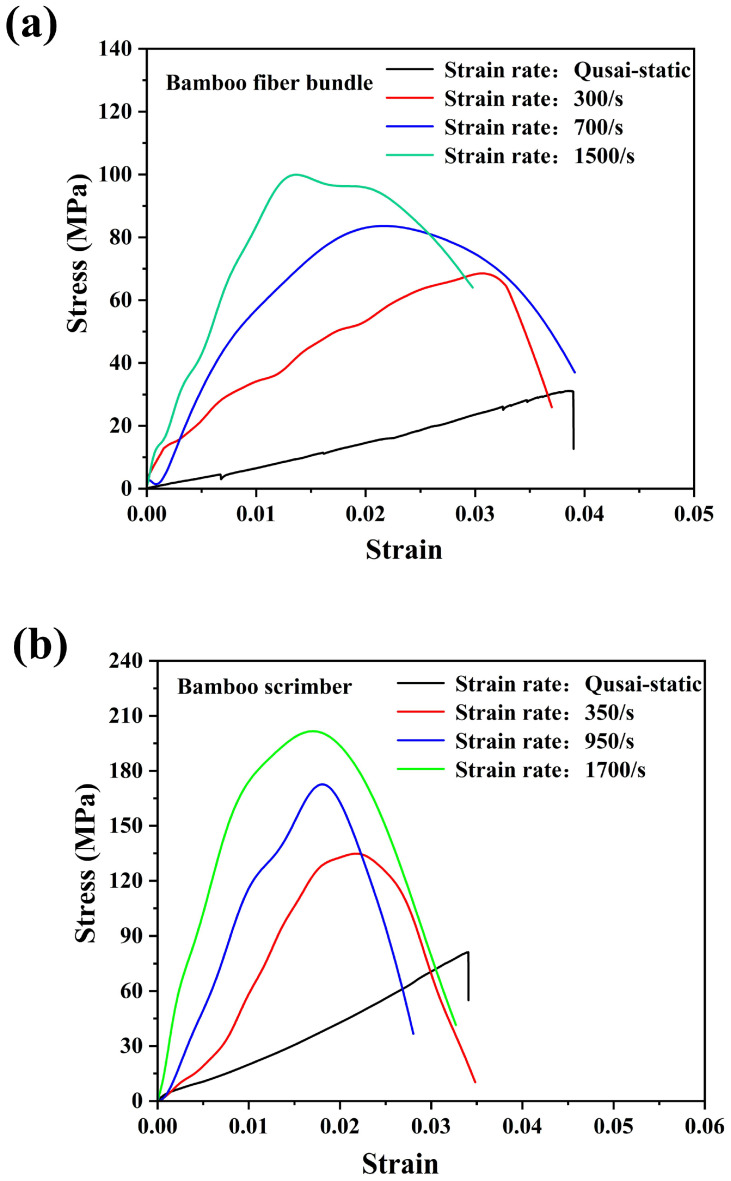
Strain rate effect on the tensile stress–strain curves of the bamboo fiber bundle (**a**) and its bamboo scrimber (**b**).

The influence of the strain rates on the tensile behavior of the bamboo fiber bundles and their bamboo scrimber are illustrated in Figure 13a and Figure 13b, respectively. To our knowledge, no published research has addressed the strain rate sensitivity of a single bamboo fiber bundle. As depicted in Figure 13a, as the strain rates increased, the bamboo fiber bundles exhibited increased brittleness, leading to fiber breakage at notably low strain levels. Meanwhile, the tensile strength of the bamboo fibers increased with the increase in strain rates. As described above, the bamboo scrimber also exhibited similar tensile behaviors. In addition, both the bamboo fiber bundles and bamboo scrimber exhibited no obvious yield stage under various strain rates. When the stress–strain curves approached the maximum stress, the testing specimens would suddenly break. This phenomenon was also found in a previous study [22].

**Figure 14:**
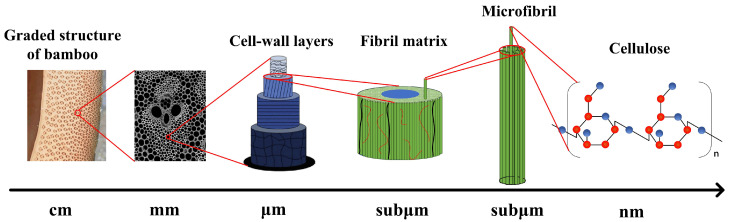
The fascinating complex hierarchical structure of natural bamboo.

Since the bamboo scrimber was mainly composed of bamboo fiber bundles and the main body of the bamboo fiber bundles was actually bamboo itself (without the green outer layer of bamboo and the yellowish inner part), we could conclude that the bamboo material had a positive strain rate effect. As shown in Figure 14, the excellent mechanical properties of bamboo can be attributed to its unique hierarchical structure. The microstructure of bamboo is mainly composed of vascular bundles and thin-walled tissue cells, exhibiting significant anisotropy [30,31,32]. Each vascular bundle has an approximately triangular structure, mainly composed of sieve tubes, pore vessels of the secondary xylem and native xylem cavities filled with thin-walled cells, fibers and basic tissues [31]. Single bamboo fibers with extremely high tensile strength can be extracted from the vascular bundle, and then elemental bamboo fibers can be further extracted from the single fibers. Finally, unit fibers can continue to extract the smallest-scale structure possessed by plant fibers—microfibrils. Like many plant materials, bamboo microfibers are mainly composed of cellulose with a long-chain molecular structure [33].

By understanding the microscopic hierarchical structure of bamboo, it is known that bamboo is composed of fibers and matrix, with fibers having high strength and modulus, while the matrix is relatively soft. This structure makes bamboo exhibit significant strain rate sensitivity during the stretching process. At high strain rates, the interaction between fibers and matrix is enhanced, and the load-bearing capacity of fibers is more significant, resulting in an increase in the stiffness and strength of the material. In addition, bamboo is mainly composed of fibers such as cellulose, which is a high polymer with a long-chain molecular structure. When subjected to tensile stress, cellulose molecular chains undergo elongation and orientation adjustment. However, when the material is subjected to high-speed tensile loads, the cellulose molecular chains will not be able to undergo stress redistribution in the testing specimen and will instead become brittle fractures, thereby improving the strength of the material.

### 3.4. Failure Mechanisms

**Figure 15:**
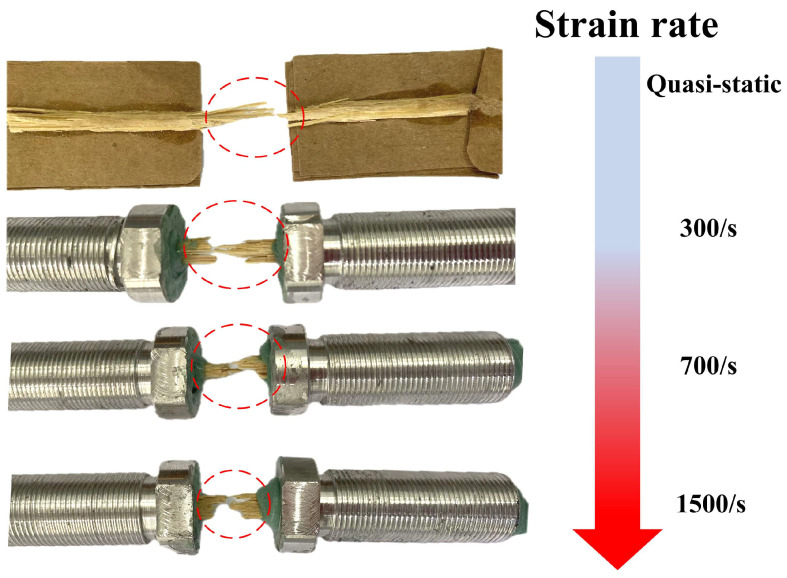
Fracture morphologies of bamboo fiber bundle under different strain rates.

Figure 15 shows the fracture patterns of bamboo fiber bundles under strain rates of quasi-static, 300/s, 700/s and 1500/s, respectively. The fracture areas are marked by red circles. It was interesting that although the strength and ductility of the bamboo fiber bundles vary significantly at different strain rates, their fracture morphologies did not exhibit significant differences. From Figure 7, Figure 8 and Figure 9, it could be seen that a single bamboo fiber bundle was actually composed of several smaller fibers combined. When the bamboo fiber bundles were subjected to tensile loads, these smaller fibers were subjected to tensile stress. When the tensile stress exceeded their ultimate strength, the fibers would fracture and fail. Although these small fibers did not break simultaneously, after the bamboo fibers ultimately failed, all fibers would undergo tensile fracture failure. Therefore, from Figure 15, it could be seen that at different strain rates, the fiber bundles exhibited similar fiber breakage failure modes.

**Figure 16:**
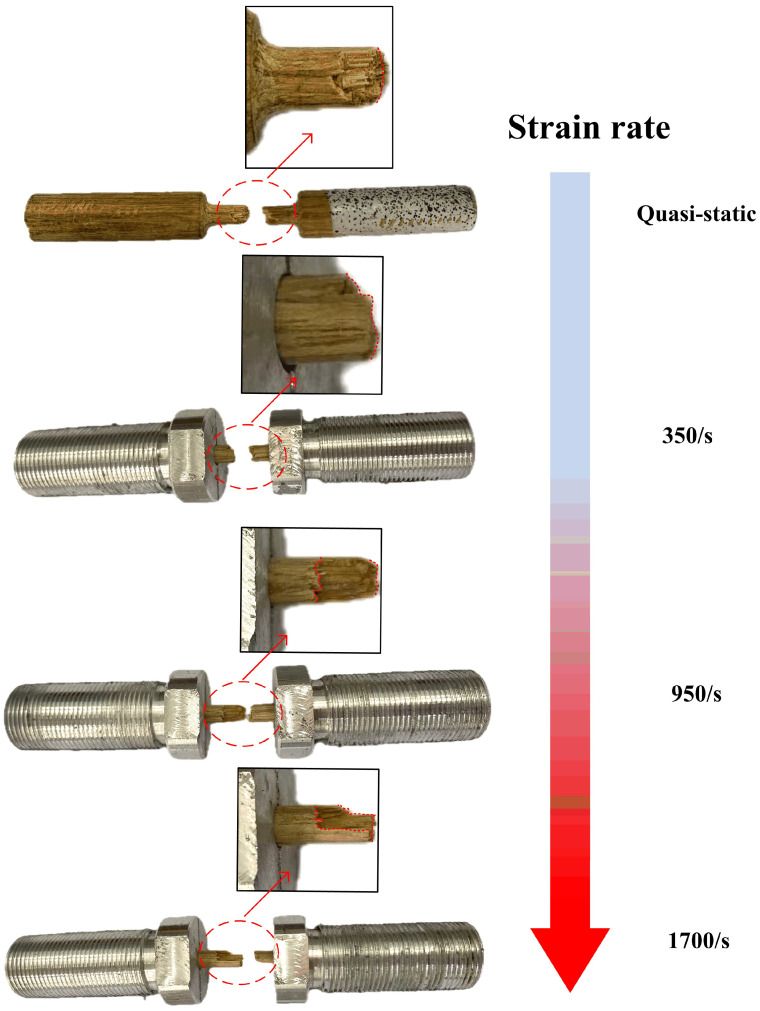
Fracture morphologies of bamboo scrimber under different strain rates.

However, as shown in Figure 16, bamboo scrimber cracks exhibited two distinct failure modes: the straight-line type and the folded-line type. For the quasi-static condition and strain rate of 350/s, the fracture surfaces of the bamboo scrimber were rough and nearly straight-line type. In comparison, under strain rates of 950/s and 1700/s, the fracture surfaces exhibited a discontinuous folded-line crack path. For fiber reinforced polymer composites (FRPCs), the interface between fibers and matrix is a critical area in composite materials, responsible for transferring loads from the matrix to the fibers [34]. Under a static load, the interface can effectively transmit stress, allowing the fibers and matrix to jointly bear the load. However, under dynamic loads, due to the extremely fast loading speed, stress transmission at the interface may not be completed in a timely manner, resulting in uneven stress distribution between the fibers and matrix. When the local stress exceeds the bonding strength of the interface, interface debonding occurs between the fiber and the matrix. Interface debonding will change the stress transmission path, causing stress to redistribute in composite materials. During the dynamic stretching process, interface debonding will expand along the fiber direction, forming a damage zone. Due to the unevenness of interface debonding and damage propagation, discontinuous line-like features appear on the fracture surface. This may explain why the failure modes of the bamboo scrimber at high strain rates were different from those at low strain rates.

## 4. Conclusions

This paper systematically studied the tensile mechanical properties and failure mechanisms of single bamboo fiber bundles and their recombinant bamboo at different strain rates through quasi-static tensile tests and dynamic tensile tests using a split-Hopkinson bar (SHTB). The main conclusions are as follows:

1. The single bamboo fiber bundle exhibited typical linear elastic behavior under quasi-static tension, ultimately leading to brittle fracture with an ultimate tensile strength of approximately 32 MPa.
2. The bamboo scrimber exhibited relatively smooth linear elastic behavior under quasi-static tension, with an ultimate tensile strength of approximately 82 MPa, which was lower than the values reported in the existing literature. This may be related to differences in sample size and the characteristics of the bamboo itself.
3. Under dynamic stretching, the ultimate tensile strength of the bamboo fiber bundles significantly increased with the increase in strain rates, showing a significant positive effect of the strain rate. For example, at a strain rate of 300/s, the ultimate tensile strength was approximately 66 MPa. At a strain rate of 1500/s, the ultimate tensile strength reached approximately 100 MPa.
4. The bamboo scrimber also exhibited a positive strain rate effect under dynamic stretching. For example, at a strain rate of 350/s, the ultimate tensile strength was about 131 MPa, which was about 60% higher than under quasi-static conditions. As the strain rate further increased to 950/s and 1700/s, the ultimate tensile strength of the reconstituted bamboo reached approximately 170 MPa and 206 MPa, respectively. The fracture strain of the recombinant bamboo at high strain rates was also smaller than that at low strain rates.
5. At different strain rates, there was no significant difference in the fracture morphologies of the bamboo fiber bundles, and the fracture zone mainly occurred at the small fiber fracture inside the fiber bundle. The fracture morphologies of the recombinant bamboo exhibited two different failure modes: linear and folded. For the quasi-static condition and 350/s strain rate, the fracture surface was rough and approximately linear. At strain rates of 950/s and 1700/s, the fracture surface exhibited a discontinuous zigzag crack path.

This study provides an important basis for a deeper understanding of the mechanical properties of bamboo fiber bundles and their composite materials under different loading conditions and may broaden the application of bamboo in engineering structures.

## Figures and Tables

**Table 1:** Ultimate stress of bamboo fiber bundle and its bamboo scrimber under different strain rates.

Bamboo Fiber Bundle	Ultimate Tensile Strength (MPa)			
Strain rate	0.002/s	300/s	700/s	1500/s
	31.66 ± 1.74	65.67 ± 3.91	84.57 ± 4.53	99.71 ± 1.43
**Bamboo Scrimber**	**Ultimate Tensile Strength (MPa)**			
Strain rate	0.002/s	350/s	950/s	1700/s
	131.33 ± 2.82	169.67 ± 3.11	206.16 ± 10.73	131.33 ± 2.82

## Data Availability

The original contributions presented in this study are included in the article. Further inquiries can be directed to the corresponding authors.
